# Activity Correlations between Direction-Selective Retinal Ganglion Cells Synergistically Enhance Motion Decoding from Complex Visual Scenes

**DOI:** 10.1016/j.neuron.2019.01.003

**Published:** 2019-03-06

**Authors:** Norma Krystyna Kühn, Tim Gollisch

**Affiliations:** 1 Department of Ophthalmology, University Medical Center Göttingen, 37073 Göttingen, Germany; 2 Bernstein Center for Computational Neuroscience Göttingen, 37077 Göttingen, Germany

**Keywords:** vision, retinal ganglion cells, direction selectivity, texture motion, feature extraction, population code, synergy, model, linear decoder, correlations

## Abstract

Neurons in sensory systems are often tuned to particular stimulus features. During complex naturalistic stimulation, however, multiple features may simultaneously affect neuronal responses, which complicates the readout of individual features. To investigate feature representation under complex stimulation, we studied how direction-selective ganglion cells in salamander retina respond to texture motion where direction, velocity, and spatial pattern inside the receptive field continuously change. We found that the cells preserve their direction preference under this stimulation, yet their direction encoding becomes ambiguous due to simultaneous activation by luminance changes. The ambiguities can be resolved by considering populations of direction-selective cells with different preferred directions. This gives rise to synergistic motion decoding, yielding more information from the population than the summed information from single-cell responses. Strong positive response correlations between cells with different preferred directions amplify this synergy. Our results show how correlated population activity can enhance feature extraction in complex visual scenes.

## Highlights

• Direction-selective ganglion cells respond to motion as well as luminance changes
• This obscures the readout of direction from single cells under complex texture motion
• Population decoding improves direction readout supralinearly over individual cells
• Strong spike correlations further enhance readout through increased synergy

## Graphical Abstract

**Figure:**
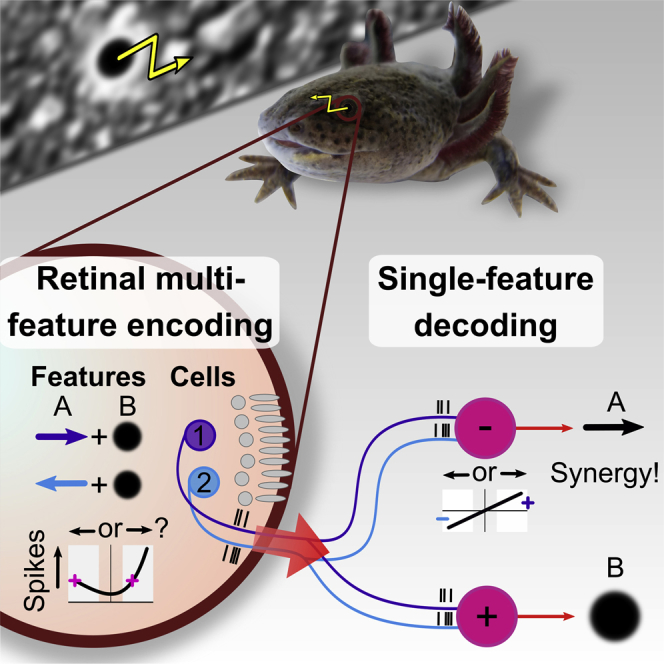

## Teaser

Direction-selective retinal ganglion cells encode visual motion but also respond to luminance changes. Kühn and Gollisch report that this dual-feature sensitivity creates ambiguities for decoding complex motion stimuli. Population decoding resolves these ambiguities and provides a synergistic motion readout.

## Introduction

A central finding for many sensory systems is that neurons are tuned to specific stimulus features, such as orientation and motion direction of visual stimuli, or pitch and spatial direction of an acoustic sound. This neuronal feature selectivity is thought to be a fundamental building block for how sensory systems parse complex sensory scenes (Barlow et al., 1964, Bizley and Cohen, 2013) and solve sensory detection tasks (Bendor and Wang, 2005, Krishnamoorthy et al., 2017, Lettvin et al., 1959, Münch et al., 2009, Ölveczky et al., 2003, Schwartz et al., 2007). In the retina, for example, direction-selective ganglion cells respond with increased activity to visual stimulus motion in a specific direction but are suppressed by motion in the opposite direction (Barlow and Hill, 1963, Borst and Euler, 2011, Lettvin et al., 1959, Mauss et al., 2017, Wei, 2018). This direction tuning is thought to support the tracking of retinal slip and the stabilization of gaze position (Sabbah et al., 2017, Yonehara et al., 2016) as well as the detection of moving objects (Marques et al., 2018, Vaney et al., 2001). How such neuronal feature selectivity contributes to sensory information processing is investigated extensively on the basis of custom-designed sensory stimuli that focus on the specific feature of interest. Direction-selective neurons in the visual system, for example, are traditionally studied with uniformly moving gratings or spots of light, where the direction of motion is varied to characterize the neurons’ directional tuning.

Yet, for most neurons that are considered as feature-selective, activity can likely also be elicited or modulated by other stimuli. Direction-selective retinal ganglion cells, for example, respond vigorously to increases or decreases in luminance inside their receptive fields, even without a motion component (Levick, 1967). Moreover, complex sensory stimuli, such as natural scenes, generally contain multiple stimulus features that vary dynamically in their strength. Under such circumstances, the response of a feature-selective neuron may be ambiguous as to whether it represents the occurrence of the particular sensory feature that is typically used to characterize it. This raises the question how downstream neurons can disentangle this ambiguity and readout information about the sensory feature in question. One possibility may be to take multiple neurons into account and consider the population code of their joint activity. For populations of direction-selective retinal ganglion cells, this can enhance the decoding of motion direction for stimuli drifting at constant speed (Franke et al., 2016, Zylberberg et al., 2016).

Here, we explored the effectiveness of population codes for resolving coding ambiguities under complex visual stimulation by focusing on recently identified direction-selective ganglion cells in salamander retina (Kühn and Gollisch, 2016). In particular, we investigated cells that are sensitive to global motion patterns, analogous to the ON direction-selective cells of the mammalian retina, which are thought to provide information about optic flow induced by head and eye movements and are vital for gaze stabilization (Osterhout et al., 2015, Simpson, 1984, Yonehara et al., 2016). We found that the single-cell encoding of textures moving with continuously varying speed and direction is curtailed by the simultaneous encoding of visual contrast. This leads to ambiguities in the readout of motion direction, but these ambiguities can be resolved by taking multiple direction-selective cells into account, leading to a synergistic population readout.

## Results

**Figure 1:**
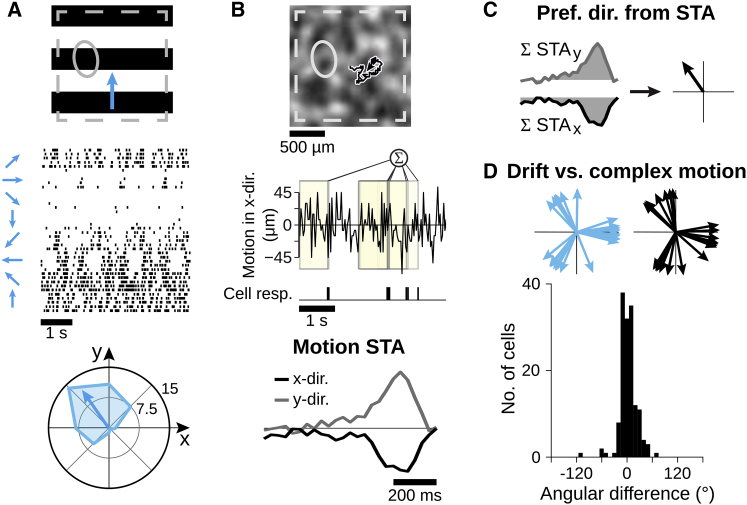
Direction-Selective Ganglion Cells Retain Their Directional Preference under Complex Texture Motion (A) (Top) Applied drifting grating for identifying direction-selective cells (ellipse: receptive field of sample cell; dashed square: recording area). (Middle) Spikes from sample cell for five trials of each of the eight grating directions. (Bottom) Mean firing rates in Hz versus motion direction. Arrow indicates preferred direction of the cell. (B) (Top) Texture for complex motion stimulus with sample trajectory. (Middle) Responses of same cell as in (A) with schematic of spike-triggered average (STA) calculation in x direction. (Bottom) STA in x and y direction. (C) Areas below STA in x and y direction are integrated to determine preferred direction for complex texture motion. (D) (Top) Preferred directions from drifting gratings (blue) and complex texture motion (black) within one sample retina (20 direction-selective cells). (Bottom) Distribution of angular differences from 149 cells with significant motion STAs from 10 retinas is shown. For responses to contrast steps and white-noise stimulation, see Figure S1.

We recorded the activity of many ganglion cells simultaneously from isolated salamander retina with multielectrode arrays and identified direction-selective cells based on their responses to drifting gratings (Figure 1A). The salamander retina contains two types of direction-selective ganglion cells (Kühn and Gollisch, 2016). One responds well to global motion stimuli, analogous to the ON direction-selective cells of the mammalian retina; the other is more sensitive to local stimulation, akin to mammalian ON-OFF direction-selective cells (Chiao and Masland, 2003, Ölveczky et al., 2003). Given that our goal here is to analyze the encoding of texture motion, we focused our analysis on the type that responds well under global motion (see STAR Methods). In contrast to the mammalian retina, these direction-selective cells are OFF type (Kühn and Gollisch, 2016; Figure S1).

### Direction Selectivity Persists under Complex Texture Motion

In order to analyze direction selectivity under complex motion, we stimulated the retina with a smoothed white-noise texture, which was shifted by small random steps (“motion steps”) in both x and y direction according to a two-dimensional random walk (Figure 1B). For assessing whether a recorded direction-selective cell responded preferentially to a specific motion pattern within this random trajectory, we calculated the spike-triggered average (STA) of the motion steps (Figure 1B, bottom). The resulting motion STAs depict the average stimulus trajectories in both x and y direction prior to the occurrence of a spike. We found that the motion STAs generally displayed a strong positive or negative peak between 150 and 200 ms prior to spiking. These peaks indicate that direction-selective cells responded asymmetrically to complex texture motion; otherwise, the independent motion steps would sum to zero. Statistical analysis by comparison with shuffled spike trains showed that the peaks in the motion STAs were significant for 75% of the analyzed direction-selective cells (n = 198 from 10 retinas), indicating directional tuning. For comparison, only 8% of non-direction-selective cells, as classified by their responses under drifting gratings, had significant peaks (n = 2,758). The reason why 25% of the direction-selective cells did not show significant peaks in their motion STAs was likely due to insufficient drive by the applied texture motion; average firing rates of these cells were low (1.5 ± 1 Hz; mean ± SD) compared to cells with significant peaks (5 ± 2 Hz).

To identify the preferred direction under texture motion of a direction-selective cell with significant motion STA, we integrated over the STA values of the x and y direction, respectively, to obtain the preferred direction as a two-dimensional vector (Figure 1C). Comparison to preferred directions obtained for drifting gratings showed a close match (angular difference 3° ± 20°; mean ± SD; Figure 1D). This indicates that direction-selective cells retain their asymmetric motion responses and preferred directions during complex texture motion.

### Motion Trajectories Can Be Decoded from Direction-Selective Cell Populations

**Figure 2:**
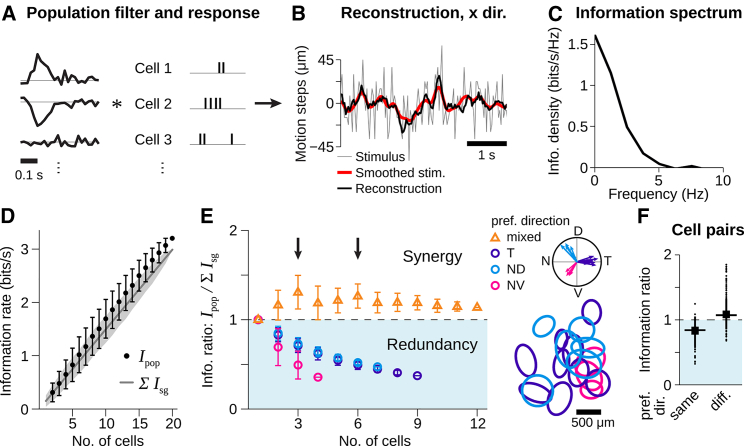
Linear Population Decoding of Random Motion Steps Is Synergistic for Direction-Selective Cells with Different Preferred Directions (A–C) Trajectory reconstruction for a population of 20 simultaneously recorded direction-selective ganglion cells. (A) Filters in x direction (left) used to transform the responses (right) into the stimulus reconstruction in (B). (B) Motion steps in x direction (gray), obtained reconstruction (black), and low-pass-filtered stimulus (red), obtained with a Gaussian kernel of 90 ms SD. (C) Spectrum of mutual information between stimulus trajectory and reconstruction. (D) Mutual information for direction-selective cell populations of different sizes. Information from population responses $I_{pop}$ (black dots) is compared to the summed information from single-cell reconstructions $\sum I_{sg}$ (gray line). Data show mean and SD (depicted by error bars and shaded area, respectively), obtained over all combinations from the 20 cells in (A)–(C). (E) Mean and SD of information ratios $I_{pop}/\sum I_{sg}$ for different subpopulations with either same preferred direction (temporal: purple; nasal-dorsal: blue; nasal-ventral: pink) or with preferred directions distributed as equally as possible across the three groups (mixed: orange). (Right) Preferred directions and receptive fields of the cells are shown. For a few cells, no receptive field was obtained. (F) Boxplots of information ratios for cell pairs. Horizontal lines and boxes indicate median and interquartile range (IQR), respectively. Vertical lines extend to data points within 1.5 × IQR, and dots indicate outliers. Data are from 10 retinas, 198 cells, 462 pairs with same and 736 pairs with different preferred directions.

How well do the responses of direction-selective cells represent the complex motion trajectory of the texture? To approach this question, we aimed at reconstructing the motion trajectory, that is, the sequence of motion steps, from population responses of direction-selective cells by employing a commonly used linear decoder model (Borst and Theunissen, 1999, Gjorgjieva et al., 2014, Warland et al., 1997). The decoder replaces each spike with an optimized filter shape for each cell and then sums the contributions from all cells (Figure 2A). This decoding scheme captures the intuitive notion of feature encoding by interpreting spikes as directly representing the presence of the feature. Similar schemes have already been successfully applied to decode contrast signals from salamander retina (Gjorgjieva et al., 2014, Warland et al., 1997). In our case, the decoder aims at reconstructing only the motion trajectory, not the contrast signals of the spatial texture. The optimal filters are obtained from a reverse-correlation analysis. They are similar in shape to the STAs in Figure 1B but are corrected for the pairwise correlations between the cells’ spike trains. For the following analyses, the filters were always obtained from the first 70% of the recording under texture motion, and the last 30% served for evaluating the reconstruction.

Comparison with the original trajectory (Figure 2B) shows that the reconstruction resembled a low-pass filtering of the stimulus motion. Fast changes in motion direction were not well captured by the reconstruction. This is not surprising, given the limited temporal resolution of phototransduction and synaptic transmission (Astakhova et al., 2015, Marre et al., 2015, Suh and Baccus, 2014, Warland et al., 1997), which is also reflected in the slow time course of the motion STAs (Figure 1B). In order to quantify the reconstruction quality, we estimated the information provided about the motion trajectory in frequency space (Figure 2C) and then obtained the total information by integrating over frequencies.

### Subpopulations with Different Preferred Directions Show Synergy in Motion Decoding

Figure 2D shows the total information $I_{pop}$ between stimulus and reconstruction for direction-selective cell populations of different sizes. As expected, taking more cells into account yielded larger information values. Yet this did not level off even for populations of up to 20 simultaneously recorded cells. For comparison, we performed the same reconstruction analysis for each individual cell and summed the resulting single-cell information values, $\sum I_{sg}$. Surprisingly, the reconstruction from population responses provided on average slightly more information than the sum of single-cell information values. Thus, the population response patterns were more informative about the motion trajectory than would be expected from the individual cells if these contributed information independently of each other, a scenario that is commonly referred to as “synergy” (Brenner et al., 2000, Gawne and Richmond, 1993, Montani et al., 2007).

For the direction-selective cells with sensitivity to global motion, which were analyzed here, preferred directions cluster into three groups, separated by 120° between each other (Figure 2E, right; Kühn and Gollisch, 2016). Thus, the observation of synergy, albeit small, might be even more surprising when considering that the larger populations had many cells with nearly the same preferred motion directions, which should encode similar aspects of the motion trajectory. To investigate the effect of having cells with similar or different preferred directions in the population, we performed the reconstruction analysis on subpopulations where all cells either had the same preferred direction or where preferred directions were distributed as equally as possible across the three groups. To focus on the relative gain or loss of information provided by the population, we normalized the information value of the population decoder by dividing through the sum of information values obtained from individual cells. Values of this ratio larger than unity correspond to synergy, whereas smaller values signify redundancy. We found that, for subpopulations with similar preferred directions, the trajectory decoding was highly redundant (Figure 2E). By contrast, decoding from subpopulations with maximal diversity in their preferred directions yielded substantial synergy, especially for subpopulations of 3 and 6 cells, where all directions were equally represented (black arrows in Figure 2E).

The synergistic information gain for these subpopulations does not simply reflect the difference in preferred directions. If each cell contributed information independently about its preferred direction, information from the population response should at most be equal to the summed information from single cells. Hence, there must be additional information about the motion trajectory in their concerted firing. But where does this additional information come from? To investigate this question, we focus in the following on analyzing pairs of simultaneously recorded cells with either different or same preferred directions. These pairs clearly show the effects of synergy and redundancy with information ratios significantly larger or smaller than unity, respectively (Figure 2F; p < 10^−3^ for both; Wilcoxon signed-rank test). Note that the information ratio can reach values of 1.5 or larger for pairs with different preferred directions, showing that more than one-third of the information about the trajectory provided by the joint activity was not available from any of the two cells alone.

### Synergistic Decoding of Motion Trajectories Generalizes over Different Textures

**Figure 3:**
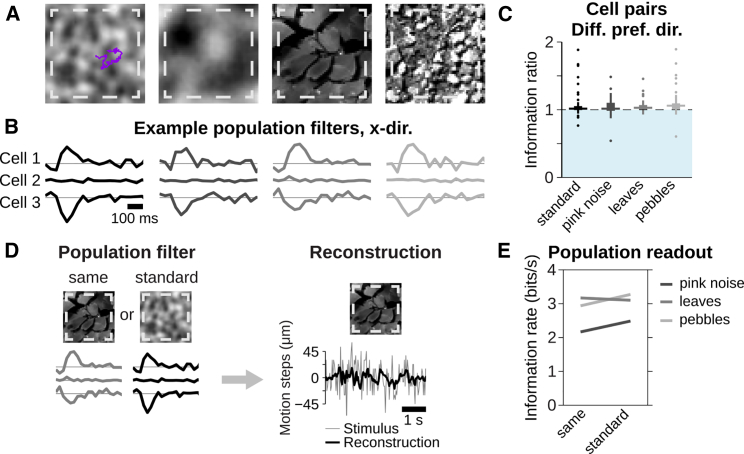
Synergistic Trajectory Readout Is Independent of the Spatial Structure of the Texture (A) Applied artificial and natural textures. From left to right: standard texture, pink-noise texture, and two natural images (“leaves” and “pebbles”) are shown. (B) Population filters in x direction of three direction-selective cells for each of the textures. (C) Information ratios of cell pairs with different preferred directions. Boxes and horizontal lines indicate IQR and median, respectively; values within 1.5 × IQR and outliers beyond are indicated by a vertical line and dots, respectively. (D) Schematic for using filters obtained from different textures for the trajectory readout. For each texture, filters were either obtained from responses under the same texture (“same”) or under the smoothed white-noise texture (“standard”). (E) Obtained information rates for different textures, depending on source of the filters. Data are from one retina, 19 cells, and 94 pairs with different preferred directions.

One may expect that the nervous system’s readout mechanisms of a motion trajectory should be independent of the specific spatial structure of the moving texture. To test this, we performed experiments where, in addition to the standard smoothed white-noise texture, a texture with a naturalistic spatial frequency spectrum as well as two natural images were applied (Figure 3A).

When examining the population readout model for each texture individually, we found that the filter shapes were reproduced across the different textures (Figure 3B). Also, pairs of direction-selective cells with different preferred directions often showed synergy (Figure 3C; p < 10^−3^ for all textures; Wilcoxon signed-rank test). The similarity of filter shapes suggests that a population readout model, obtained from one texture, should generalize across textures. To test this, we compared the reconstruction of a motion trajectory for a given texture based on population filters that were either obtained from responses to the same texture or from responses to the standard texture (Figure 3D). Even though the total information obtained from a direction-selective cell population about the motion trajectory varied for the different textures, the information values were similar regardless of whether filters came from the same texture or from the standard texture (Figure 3E), and information readouts from pairs with different preferred directions were generally synergistic (p < 0.005 for all textures). Thus, the population readout model indeed generalizes across textures.

### Noise Correlations Are Not Essential for Synergistic Decoding of Motion Trajectories

**Figure 4:**
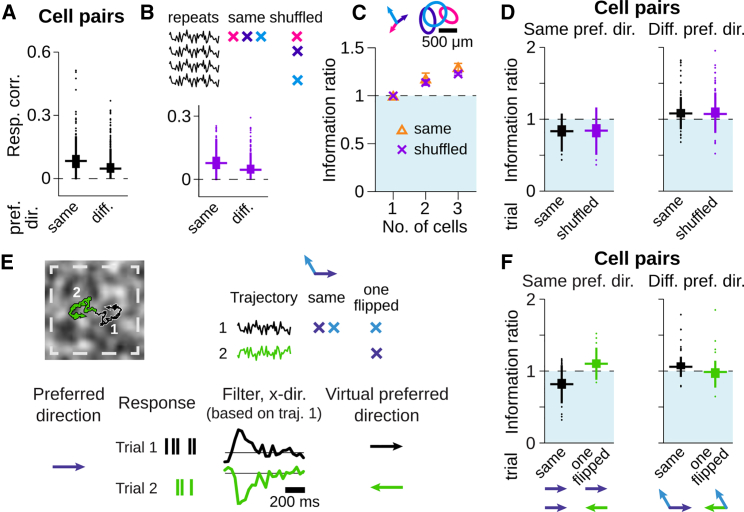
Synergy and Redundancy Do Not Depend on Noise Correlations and Rely on Relative Directional Preference (A and B) Response correlations between cell pairs with same or different preferred directions. Responses are taken from same (A) or “shuffled” trials (B) of repeated stimulus presentations (see inset). Data are from 6 retinas, 104 cells, 389 pairs with same and 615 pairs with different preferred directions. (C) Information ratios for three sample cells with different preferred directions and nearby receptive fields (top) from same or “shuffled” trials. Mean and SD are from all eligible trial combinations. (D) Information ratios of cell pairs with same (left) or different preferred directions (right) from same (black) or shuffled trials (purple). Data are from 6 retinas, 104 cells, 325 pairs with same and 501 pairs with different preferred directions. (E) Schematic for “flipping” a cell’s preferred direction. Trajectory of second trial (green) was flipped along the x and y axis. Responses of cell pairs were either combined from the same trial (“same”) or from different trials (“one flipped”). (Bottom) Sample responses, filters, and corresponding preferred direction of a rightward-motion-preferring cell are shown. (F) Information ratios of cell pairs with same (left) and different preferred directions (right), with reconstructions obtained from filters and responses to same trials (black) or different trials so that one preferred direction is flipped (green). Data are from one retina, 21 cells, 110 pairs with same and 94 pairs with different preferred directions. Boxplots indicate median (horizontal line) and IQR (box); values within 1.5 × IQR and outliers beyond are indicated by a vertical line and dots, respectively.

Synergy and redundancy in neuronal population codes have often been linked to correlated activity (Averbeck et al., 2006, Panzeri et al., 1999, Schneidman et al., 2003). We therefore checked the spike correlations for pairs of direction-selective ganglion cells during random texture motion, based on spike counts in 33-ms windows, corresponding to the stimulus update rate. For cells with different preferred directions, activation of one cell by motion in its preferred direction should be accompanied by suppression of the other by a motion component in its null direction, which might suggest negatively correlated activity. Nonetheless, pairwise response correlations between direction-selective cells were always positive, even for cells with different preferred directions (Figure 4A).

The observed correlations could reflect signal correlations, resulting from stimulus-induced co-activation, as well as noise correlations, which may follow from coupling or shared input noise. Noise correlations are often considered as a potential source of synergy and redundancy, in particular through spike synchronization (Averbeck et al., 2006, Schneidman et al., 2003). We therefore investigated the effect of noise correlations by recording responses to repeated trials of identical texture motion. We then compared the decoding performance when cells responded to the same trial with the performance of the same cells after shuffling the trials for each cell, which removed potential noise correlations.

Although this shuffling reduced the overall correlations by a small yet significant amount for pairs with different as well as same preferred directions (Figure 4B; Wilcoxon signed-rank test: p < 10^−3^ in both cases), it did not influence the patterns of synergy and redundancy. For a sample population with three cells of different preferred directions and strongly overlapping receptive fields, Figure 4C shows that shuffling the trials had hardly any effect on the decoding performance; in particular, it did not abolish synergy. Analyzing all pairs of simultaneously recorded direction-selective cells revealed that the distributions of information ratios for cell pairs with different or same preferred directions did not change significantly when potential noise correlations were removed (Figure 4D; Wilcoxon signed-rank test: p = 0.20 and p = 0.70, respectively). Hence, direction-selective ganglion cells might show noise correlations (though slow fluctuations in the population activity may also contribute to the reduced correlations after shuffling; see Brody, 1999), but these do not account for the observed synergy or redundancy.

### Virtual Flip of One Preferred Direction in a Cell Pair Can Turn Redundancy into Synergy

We next asked whether the emergence of synergy can be explained by how individual direction-selective cells encode complex texture motion. If this is the case, then flipping the preferred direction of one cell in a cell pair should exchange synergy for redundancy and vice versa because the flipping reverses whether two cells have similar or opposing preferred directions. We can simulate this situation by showing the same moving texture twice but once with the trajectory flipped for both x and y direction (Figure 4E, top). Thus, we could compare the normal trajectory decoding to the decoding where one cell responded to the flipped trajectory, which effectively reversed the preferred motion direction of this cell (Figure 4E, bottom).

This indeed affected synergy and redundancy in the hypothesized way. For cell pairs with originally similar preferred directions, redundancy was turned into synergy, consistent with the now opposing preferred directions (Figure 4F, left). For direction-selective cell pairs with different preferred directions, on the other hand, synergy was essentially abolished by flipping one cell’s preferred direction (Figure 4F, right). Note, though, that for such cell pairs preferred directions were originally not diametrically opposing but rather separated by about 120° so that, after the switch, there was still an angular difference of about 60°. This explains why the effect is smaller than for cell pairs with the same preferred direction. These results demonstrate that the observed synergistic population readout arises from how individual direction-selective cells encode complex texture motion.

### Individual Direction-Selective Cells Show Ambiguities in Direction Encoding

**Figure 5:**
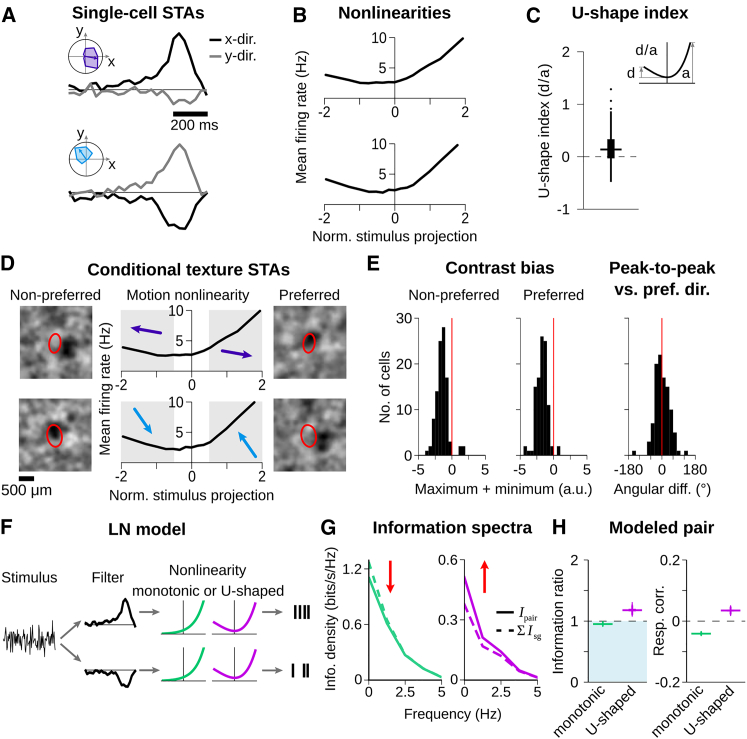
Non-monotonic Motion-Response Relations Can Induce Synergy for Cell Pairs with Opposing Directional Preference (A and B) STAs in x and y direction (A) and nonlinearities (B) of two direction-selective cells. Insets show directional tunings to drifting gratings. (C) U-shape index of nonlinearities of the same 149 cells as in Figure 1D. Boxes and horizontal lines indicate IQR and median, respectively; values within 1.5 × IQR and outliers beyond are indicated by a vertical line and dots, respectively. Inset shows schematic of U-shape index calculation. (D) Conditional texture STAs for non-preferred (left) and preferred motion trajectories (right) of same cells as in (A). (E) Histograms of contrast biases of conditional texture STAs for non-preferred (left) and preferred motion trajectories (middle) and of angular differences (right) between the preferred direction of a cell and the vector connecting the negative peaks in the two conditional texture STAs. Data are from 10 retinas and 102 cells. (F) LN model for simulating responses of cell pairs, using one-dimensional stimulus filters from cells in (A) and either monotonic (green) or U-shaped nonlinearities (purple), both obtained from fits to measured nonlinearities that correspond to one-dimensional motion (Figure S2). Spike trains were generated by a Poisson process. (G) Information from pair responses (solid line) was decreased for monotonic nonlinearities (left) and increased for U-shaped nonlinearities (right) compared to summed single-cell information (dashed line), indicating redundancy and synergy, respectively. (H) Boxplots as in (C) of information ratios and response correlations from 1,000 simulation runs. For model of two-dimensional motion, see Figure S2.

To better understand the response characteristics of individual direction-selective cells under complex texture motion, we analyzed their responses in the framework of the linear-nonlinear (LN) model. This model relates the incoming stimulus—here, the random sequences of motion steps in x and y direction—to the firing rate of a neuron by first convolving the stimulus with a linear filter and then applying a nonlinear transformation (the model’s “nonlinearity”) to obtain a firing rate. For the Gaussian white-noise statistics of the motion steps applied here, the cell’s motion STA, as measured in Figure 1B, can be used as the filter of the LN model (Chichilnisky, 2001). Note that the separately displayed STAs for x and y direction (Figure 5A) correspond to two components of a single stimulus filter. The nonlinearity can then be obtained by applying this filter to the stimulus and compiling a histogram of the average measured firing rates for given ranges of the filter output (Figure 5B).

Surprisingly, we found that direction-selective cells had non-monotonic, U-shaped nonlinearities in their response to complex texture motion (Figures 5B and 5C). This means that direction-selective cells do not only show responses to motion into their preferred direction but also respond to motion stimuli in the opposite direction, which corresponds to the null direction under drifting gratings. Thereby, the encoding of motion direction by individual direction-selective cells during complex texture motion is ambiguous. We quantified the degree of non-monotonicity of direction-selective cells by introducing a U-shape index, which is negative for monotonic nonlinearities and positive for a non-monotonic U-shape. The U-shape index was generally larger than zero, indicating that nonlinearities were often non-monotonic (Figure 5C; Wilcoxon signed-rank test: p < 10^−3^). We also analyzed how responses of direction-selective cells were affected by motion orthogonal to the preferred direction and found that such motion modulates the firing rate via an approximately symmetric U-shaped nonlinearity (Figure S2). Thus, motion in any direction in the two-dimensional plane can activate the direction-selective cell to some extent.

To investigate the origin of the U-shaped nonlinearities, we asked whether the OFF-type contrast sensitivity of the cells was relevant for responses both to motion in the preferred as well as in the non-preferred direction or whether responses to non-preferred motion rather involved ON-type contrast signals, which could be consistent with specific models of direction selectivity (Behnia et al., 2014, Clark et al., 2011). We therefore computed STAs of the spatial stimulus patterns separately for spikes associated with preferred and non-preferred motion, that is, spikes contributing to the right and left portions of the U-shaped nonlinearities, respectively. Although these conditional STAs exhibit considerable structure inherited from the correlations in the textured stimulus, we found that a prominent dark spot typically appeared near the cell’s receptive field in both cases (Figure 5D). The exact location of the dark spots relative to the receptive field outlines seems to be somewhat variable, owing to stochastic noise both in the estimation of the conditional STA and in the receptive field location. Nonetheless, a systematic spatial arrangement of the dark spots can be deduced from their relative positions: we found that, compared to the dark spot for the preferred direction, the location of the spot for the non-preferred direction was generally shifted along the cell’s preferred direction (Figure 5E). This indicates that it is dark spots starting to enter the receptive field from either side that are responsible for the two arms of the U-shaped nonlinearities, in line with the OFF-type contrast sensitivity of these cells.

### Opposing Direction Preferences and U-Shaped Nonlinearities Can Induce Synergy

We hypothesized that the ambiguity in the single-cell encoding of motion direction represented by the U-shaped nonlinearity is critical for the observed synergy in populations of direction-selective cells. To test this hypothesis, we simulated the responses for a pair of direction-selective cells with an LN model, allowing us to alter the shape of the nonlinearity. The goal of the model was to investigate how this shape may affect the decoding of a stimulus parameter from cell pairs with opposite tuning. Thus, rather than describing how a cell might respond to motion as well as luminance signals in the texture, we used a phenomenological model in which, for simplicity, the only input was a sequence of motion steps in one dimension. The two modeled cells independently filtered the sequence with opposing filter shapes, taken from the experimentally obtained motion STAs (Figure 5A). The filtered signals were transformed into firing rates by applying the same nonlinearity for both cells, which was either monotonic or U-shaped (Figure 5F). Spikes were then generated by a Poisson process.

Trajectory reconstructions and information ratios were obtained in the same way as for the experimental data. We found that the shape of the nonlinearity indeed has a strong effect. For monotonic nonlinearities, the information obtained by the two-cell decoder is reduced compared to the sum of single-cell information values, whereas for U-shaped nonlinearities, the information from the two-cell decoder is relatively increased (Figures 5G and 5H). This also holds for a model of two-dimensional motion (Figure S2). Thus, non-monotonic U-shaped nonlinearities of stimulus encoding can turn an otherwise redundant readout from cell pairs into a synergistic readout. An essential component for this synergy is that information transmission of single cells is compromised by the U-shaped nonlinearity, yielding information values about three times smaller than in the case of the monotonic nonlinearities. This provides the opportunity for synergistic improvements through the multi-cell decoder by resolving the single-cell ambiguities.

### Response Correlations from Shared Local Visual Stimulation Enhance Synergy

The U-shaped nonlinearities may also explain the positive correlations between direction-selective cells with different preferred directions (Figure 4A). The non-monotonic shape indicates that strong motion signals, regardless of direction, tend to increase the activity of all direction-selective cells, leading to some level of co-modulation. Indeed, the investigated models show that U-shaped nonlinearities can induce positive response correlations (Figures 5H and S2D). Essentially, this co-modulation occurs because strong motion signals provide the potential for simultaneous darkening of different receptive fields (Figures 5D and 5E). Yet, for two given cells, only some of these motion events will provide actual darkening for both receptive fields, which limits the strength of the positive correlations. If, however, the two receptive fields are close to each other or even overlapping, they should experience similar patterns of brightening and darkening, which should then strengthen the cells’ correlations.

**Figure 6:**
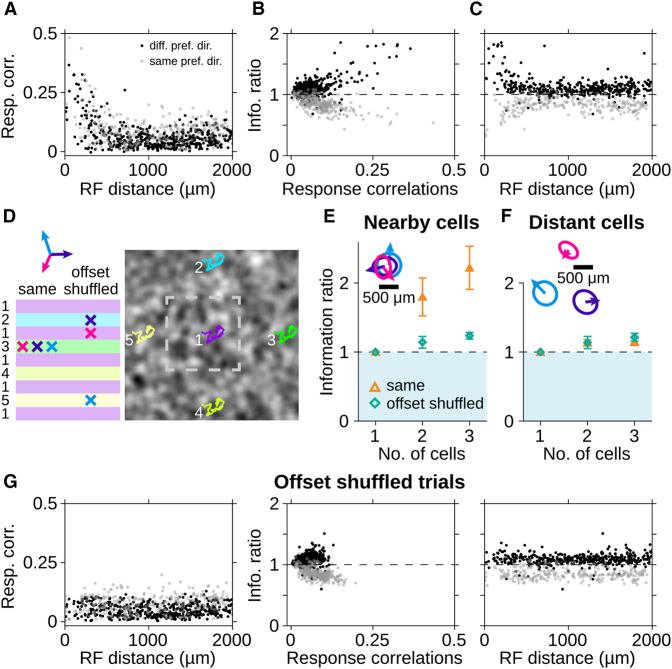
Response Correlations in Pairs of Direction-Selective Cells Enhance Synergy and Redundancy in Motion Decoding (A–C) Relations between response correlation and receptive field distance (A), between information ratio and response correlation (B), as well as between information ratio and receptive field distance (C) for cell pairs with different (black) or same preferred directions (gray). (D–G) Influence of local stimulus structure on population decoding. (D) Schematic of “offset shuffling.” Information ratios and response correlations were either obtained from cell responses within the same trial (same) or different trials with different offsets (“offset shuffled”). (E and F) Examples of mean information ratios and SDs obtained over all trial combinations of three direction-selective cells with different preferred directions with nearby (E) or distant receptive fields (F). (G) Same as (A)–(C) but from offset shuffled trials. Data points represent mean over all trial combinations. Data are from 5 retinas, 92 cells, 315 pairs with same and 407 pairs with different preferred directions.

Indeed, we found that the positive response correlations were particularly pronounced for pairs of direction-selective cells whose receptive fields were spatially close to each other (Figure 6A). For cell pairs with the same preferred direction, stronger positive correlations generally led to more pronounced redundancy (Figure 6B). This is expected because the correlations imply that cells respond to the same parts of the stimulus trajectory. However, in contrast to this expectation, cell pairs with different preferred directions displayed larger synergy when their responses were more strongly correlated (Figure 6B). It follows that synergy is particularly strong for cell pairs with different preferred directions and nearby receptive fields (Figure 6C).

In order to test whether the elevated correlations of nearby direction-selective cells resulted from experiencing nearby parts of the texture, we aimed at creating a virtual shift of individual receptive fields: we repeated a 15-min trajectory with different spatial offsets (Figure 6D) and compared the normal trajectory reconstruction to a reconstruction where responses of different cells were taken from different, spatially offset trials. For a sample set of three cells with different preferred directions and strongly overlapping receptive fields, we found that this virtual receptive field shift considerably reduced the originally large synergy but did not abolish it completely (Figure 6E). In fact, the synergy of this sample set was now in a similar range as for three sample cells whose receptive fields were distant from each other (Figure 6F). For this second sample set, the original synergy was much smaller, but was not further reduced by the spatial offset. These findings were corroborated by population analysis of cell pairs (Figure 6G). The spatial offset abolished the effect that particularly strong correlations and synergy occurred for nearby direction-selective cells but otherwise had little effect on the baseline of correlations, synergy, and redundancy. This indicates that the elevated correlations and synergy values of nearby direction-selective cells indeed result from shared local stimulation, so that motion-induced luminance changes inside the two receptive fields are correlated in sign and strength.

### Direction-Selective Cells with Different Preferred Directions Are Anti-correlated in Their Motion-Response Patterns

The contrast-induced co-modulation of activity might conceal correlations that are linked to motion direction. We therefore analyzed how the joint responses of pairs of direction-selective cells are related to sequences of motion steps by applying canonical correlation analysis (CCA) (Hotelling, 1936). CCA can be viewed as an extension of standard reverse-correlation techniques by correlating stimulus features with more complex response patterns than single spikes, including patterns extended over time and over multiple cells. The technique identifies combinations of response patterns and corresponding stimulus features that are most strongly correlated with each other (Macke et al., 2008).

**Figure 7:**
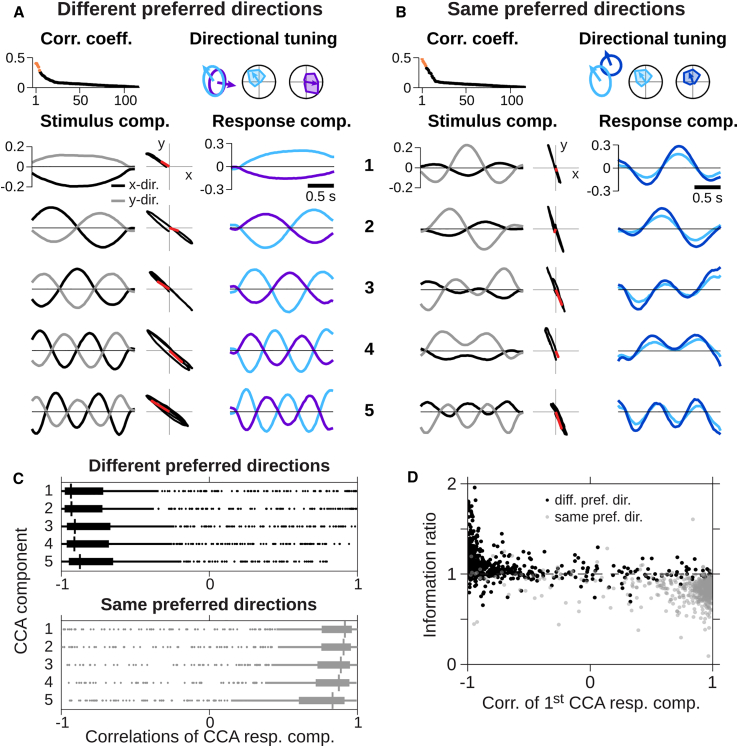
Motion-Related Responses of Cell Pairs with Different Preferred Directions Are Anti-correlated (A and B) Canonical correlation analysis (CCA) of responses from sample pairs with different (A) or same preferred directions (B). (Top) Correlation coefficients of CCA (left) and cell properties (right) are shown. (Below) First five CCA stimulus components (left), their projection onto x-y plane (middle, red arrows indicate first motion step), and corresponding response components (right, colors indicate cells above) are shown. (C) Boxplots of correlation coefficients of first five response components of all pairs with different (top) or same preferred directions (bottom). Boxes and horizontal lines indicate IQR and median, respectively; values within 1.5 × IQR and outliers beyond are indicated by a vertical line and dots, respectively. (D) Correlation coefficients of first response component in relation to information ratios of pairs with different (black) or same preferred direction (gray). Data are from 10 retinas, 198 cells, 462 pairs with same and 736 pairs with different preferred directions.

To identify response patterns that best correlate with motion signals of the applied moving texture, we used 2-s-long segments of the motion trajectory together with the corresponding responses, composed of the spiking activity of two direction-selective ganglion cells over the 2 s. CCA then provided an array of motion trajectory segments and corresponding response components of the two cells, ordered by the strength of their correlation (see Figures 7A and 7B for two examples). For a sample pair of direction-selective cells with different preferred directions (Figure 7A), the stimulus-response correlations were strongest for sustained texture motion either in the negative x direction and positive y direction or vice versa, as the overall sign of the obtained components is undetermined. The corresponding response component shows that, depending on the actual direction of this motion component, either one or the other of the two cells responded with increased activity. Thus, this motion component was in fact associated with anti-correlated activity of the two cells. Similarly, the next four response components, which corresponded to motion at higher temporal frequencies, were characterized by anti-correlated activity at those higher frequencies. For a sample pair of direction-selective cells with the same preferred direction, on the other hand, the response components that were most strongly related to motion components all showed positively correlated activity patterns (Figure 7B).

Population analysis corroborated these findings. For each recorded pair of direction-selective cells, we selected the first five response components obtained by CCA and computed the correlation between the activity profiles of the two cells. For cell pairs with different preferred directions, this revealed strongly negative correlations in the response patterns of the two cells, whereas for cell pairs with the same preferred direction, the response pattern correlations were generally positive (Figure 7C). Furthermore, the largest synergy values occurred for cell pairs with particularly strong negative correlations in the first response component of the CCA (Figure 7D).

The anti-correlated activity patterns of the CCA for cell pairs with different preferred directions might appear to be at odds with the overall positively correlated activity (Figures 4A and 6A). However, the CCA identifies such response patterns that are associated with specific motion features. Thus, we can interpret the overall positive spike correlations as resulting from common activation by luminance signals, which can occur unspecifically for motion in any direction; specific motion sequences, on the other hand, are represented by response patterns that are anti-correlated between cells with different preferred directions.

### A Subtractive Code of Direction-Selective Cells with Different Preferred Directions Shows Synergy along the Axis of Motion Opponency

The finding that cell pairs with different preferred directions show positive overall activity correlations but negative correlations in their motion-related response components suggests that a good readout strategy for motion information might be to subtract the activity of the two cells. This should reduce the activity induced by common contrast changes and highlight the motion-related activity. For cell pairs with the same preferred direction, on the other hand, the sum rather than the difference should be informative about the motion trajectory, because such cells showed positive correlations in the CCA response components. Note, however, that the summed activity does not help disambiguate motion-related from contrast-related responses; thus, no synergy is expected from summed responses.

**Figure 8:**
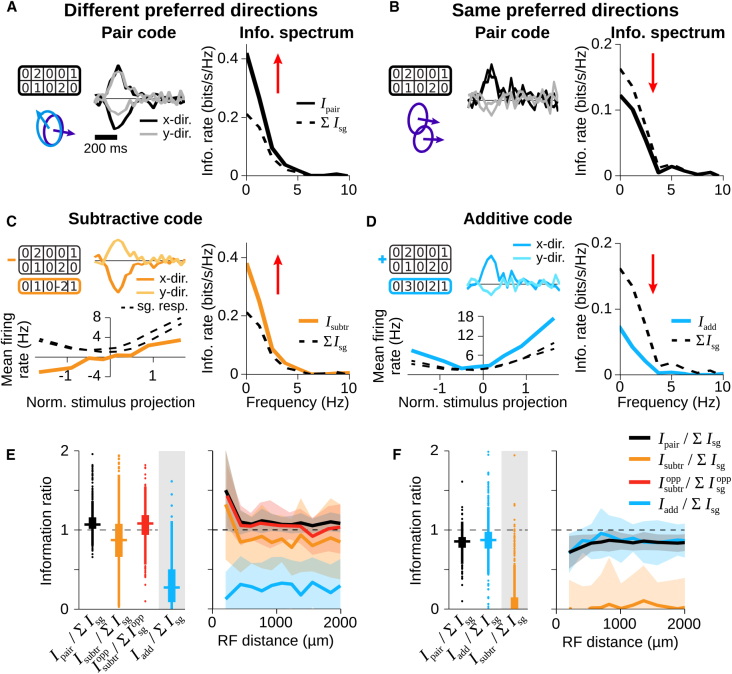
Subtractive Code of Cell Pairs with Different Preferred Directions Captures Synergy (A and B) Population codes for sample pairs with different (A) or same preferred directions (B). (Left) Receptive fields, preferred directions, and pair filters are shown. (Right) Information spectrum of the population code (solid line) compared to the summed single-cell information spectra (dashed) is shown. (C) Subtractive code of cells in (A). Response filter and nonlinearity (left) as well as information spectrum (right) of subtractive code (orange), together with nonlinearities and summed information from single-cell responses (dashed) are shown. (D) Same as (C) but for summed responses (blue) of cells in (B). (E and F) Information ratios as boxplots (left; horizontal line: median, box: IQR, vertical line: values within 1.5 x IQR, dots: outliers beyond 1.5 × IQR) and binned by receptive field distance (right; thick line indicating mean; shaded region SD) from pair responses (black); subtractive code (orange); subtractive code along either x or y direction, depending on which showed greater motion opponency (red); and additive code (blue) for cell pairs with different (E) or same preferred directions (F). Data are from same pairs as in Figures 7C and 7D.

The sum or difference of two spike trains reduces the joint response patterns to a single activity sequence. To test whether such a reduced code could capture the transmitted information, we analyzed linear decoders that take as input either the difference or sum of the binned spike counts of two cells. For a sample cell pair with different preferred directions (Figure 8A), a subtractive code led to information rates that were larger than the summed single-cell information rates (Figure 8C, right), thus capturing the synergistic decoding of the original two-cell decoder (Figure 8A). Furthermore, the nonlinearity of an LN model for the response difference turned out to be monotonic (Figure 8C, left), indicating that the ambiguities of motion decoding reflected in the U-shaped nonlinearities of individual cells were resolved by using the response difference. For a sample pair of direction-selective cells with the same preferred direction, on the other hand, we found that the additive code had a similar performance as the two-cell decoder (Figures 8B and 8D).

These findings were corroborated by population analysis (Figures 8E and 8F). For cell pairs with different preferred directions, the subtractive code (Figure 8E, orange) generally captured a large portion of the information contained in the joint response patterns, whereas the additive code (Figure 8E, blue) was considerably worse. Note that the somewhat reduced performance of the subtractive code compared to the two-cell decoder is expected because the two preferred directions are generally not diametrically opposed but separated by only about 120°. Thus, there is a motion axis for which the two cells had common directional preference, and the subtractive code should be deleterious along this axis. However, when focusing the analysis on a one-dimensional motion axis for which two cells had opposed preference (Figure 8E, red), the performance of the subtractive code again reached similar levels of synergy as the full two-cell decoder. For cell pairs with the same preferred direction (Figure 8F), on the other hand, it was the additive code that performed as well as the joint responses.

## Discussion

Neurons in the visual system are often regarded as feature encoders whose responses are tuned to the strength of a certain visual feature appearing within a cell’s receptive field. During natural viewing, however, there may be multiple features that simultaneously affect a cell, e.g., contrast, spatial pattern, motion speed, and direction (Deny et al., 2017, Im and Fried, 2016). Here, we asked how information about a single feature, motion direction, can be extracted from the multiplexed signaling of direction-selective retinal ganglion cells under complex visual stimulation. Information about motion direction is vital for stabilizing the gaze during natural viewing (Simpson, 1984, Yonehara et al., 2016) and for coping with motion-induced image blurring under fixational eye movements (Pitkow et al., 2007, Rucci et al., 2007). For such tasks, the neural code for motion direction should ideally not be influenced by other stimulus components, such as the spatial pattern of the moving scene. Direction-selective ganglion cells, however, are not only tuned to motion direction but concurrently respond to luminance changes in their receptive fields, which complicates the readout of motion direction from individual cells.

### Synergistic Motion Readout Is Based on Resolving Ambiguities in Stimulus Encoding

We found that the readout of motion direction from individual direction-selective cells under complex texture motion is ambiguous; spikes could either signify motion into the preferred direction or preferred contrast brought into the receptive field. Hence, for separating information about motion direction and contrast, signals from further cells are required (da Silveira and Berry, 2014). A cell with a different preferred direction will then not only contribute information about its own preferred direction but also provide additional information by separating motion from contrast signals. This additional information leads to the observed synergy in the readout from cells with different preferred directions.

The underlying mechanism for this synergy is that, during periods of co-activation of two cells, their activity difference extracts motion-related information by reducing the effect of confounding luminance-triggered signals. The co-activation is stochastic for cell pairs with distant receptive fields but becomes more systematic and stronger for nearby cells, which makes the cancelation of these luminance signals more effective and can thereby lead to stronger synergy. Interestingly, this mechanism is similar to a phenomenon known as the “sign rule,” which states that noise correlations have beneficial effects on decoding when they have opposite sign compared to the signal correlations (Averbeck et al., 2006, Hu et al., 2014, Jeanne et al., 2013, Oram et al., 1998, Panzeri et al., 1999). In the present case, motion direction is signaled by the spiking difference of two neurons (negative signal correlations), and therefore, co-modulation of their signaling (positive “noise” correlations) would increase the reliability of the signal readout. And although actual noise correlations do not play a role here, the contrast-induced activity essentially acts as noise in the context of motion decoding. In accordance with the sign rule, the co-activation has a beneficial effect on decoding the negatively correlated motion signals of cells with different preferred directions and a negative effect on decoding the positively correlated motion signals of cells with the same preferred direction.

We here used an optimal linear decoder (Warland et al., 1997) to estimate motion direction from the cells’ responses. The decoder recovers a linear filter for each cell, which can be regarded as the encoded feature. In this way, strong responses correspond to more of this feature and weaker responses correspond to less of it. This resembles our general intuition of how a feature encoder provides information about the corresponding feature.

What are the limiting factors for the reconstruction performance and the retrieved information rates? First, the low-pass filtering of the cells does not allow for reconstruction of high-frequency components of the trajectory. Second, motivated by the scenario of fixational eye movements, the jittering trajectory has relatively small net-motion, typically translating the texture by only around 150 μm per second. This is less than half the typical receptive field diameter of the investigated cells (Kühn and Gollisch, 2016), providing for a challenging reconstruction task. Finally, and most importantly, the retrieved information rates are curtailed by the observed inherent coding ambiguity with respect to motion direction. The U-shaped nonlinearities limit the directional information, as can be seen by the three-fold larger single-cell information rates of a comparable model with monotonic nonlinearities (Figure 5G). Note also that the decoder reflects how spikes represent motion direction but does not aim at capturing all of the information in the spike trains about the stimulus. For example, a completely symmetric nonlinearity would lead to zero information retrieved from the linear reconstruction. This would be consistent with the complete lack of any directional information, but spikes would still be informative about whether or not substantial motion occurred in any direction.

Alternative decoders, for example based on maximum likelihood or Bayesian approaches, which have been used for decoding the motion direction of uniformly drifting bars (Fiscella et al., 2015, Pouget et al., 2000, Zylberberg et al., 2016), might retrieve larger information values. Interestingly, however, it has been suggested that the decoding performance of these models is similar to an optimal linear decoder for drifting bar stimuli (Fiscella et al., 2015). Nonlinear extensions of the optimal linear decoder, using regularization in the filter estimation (Botella-Soler et al., 2018, Maheswaranathan et al., 2018, Marre et al., 2015), might also improve the performance of both single-cell and population readout. Nevertheless, these extensions by themselves will not resolve the ambiguities about motion and contrast information contained in the single-cell responses, as these are a generic feature of how the direction-selective ganglion cells respond to complex texture motion.

### Population Signals May Allow Purpose-Related Feature Readout in Different Projection Areas

Direction-selective ganglion cells are thought to contribute essential information about eye and head movements to downstream brain areas. In mice, they are known to project to different target regions in the brain, e.g., the nodes of the accessory optic tract, superior colliculus, and lateral geniculate nucleus (Dhande et al., 2013, Dhande et al., 2015, Gauvain and Murphy, 2015). Because individual retinal ganglion cells often have more than one projection target (Ellis et al., 2016), each direction-selective ganglion cell might contribute to multiple readout schemes so that different features can be extracted from their multiplexed signaling.

For pairs of direction-selective cells with different preferred directions, we found that motion encoding ambiguities can be largely resolved by decoding their spiking difference. For a biological implementation of such a subtractive-code readout, a downstream neuron would simply have to receive excitation from one direction-selective cell and inhibition from the other. Such a circuit had already been proposed by Levick et al. (1969) as a way to sharpen directional tuning. Because retinal ganglion cells are generally excitatory, inhibition should come through a downstream interneuron, which would then also be directionally tuned. Indeed, the optic tectum of zebrafish was found to contain two populations of oppositely tuned direction-selective neurons, which receive excitatory as well as inhibitory directionally tuned inputs (Gabriel et al., 2012). The excitatory inputs originated from direction-selective retinal inputs to the superficial layers of the tectum. The inhibitory inputs seemed to emerge from direction-selective neurons of the other tectal population and were tuned to non-preferred directions of their recipient neurons. For the applied drifting bars, the inhibitory inputs appeared to have little effect on the directional tuning of the tectal neurons. Yet, based on our findings here, we predict that, during more complex visual motion stimulation, the inhibition should cancel contrast-induced activity and thus provide a more direct representation of motion direction.

An additive code of direction-selective ganglion cells, on the other hand, might be implemented in the mouse lateral geniculate nucleus, where neurons have been found that receive direct input from several direction-selective ganglion cells (Rompani et al., 2017). This additive code might contribute to separating the global motion signal in the visual stimulus from local image information. Hence, depending on the biological implementation of signal integration in the corresponding target area, diverse features can be read out from the joint signals of multiple direction-selective retinal ganglion cells.

For direction-selective ganglion cells with different preferred directions, we have here considered a two-cell subtractive code. Yet, it is straightforward to extend this decoding scheme to more neurons, in particular, for taking into account the three groups of different preferred directions. Analogous to a population-vector-type readout (Georgopoulos et al., 1986, Salinas and Abbott, 1994), motion information about an arbitrary direction could be extracted by weighing each cell’s activity with the cosine of the angular difference between the direction of interest and the cell’s preferred direction. Effectively, the three groups of different preferred directions in this system of direction-selective ganglion cells can thus be considered as a minimal set required for disentangling the three stimulus variables that affect the cells’ responses, namely motion in x direction, motion in y direction, and local luminance changes.

## STAR★Methods

### Key Resources Table

**Table:** 

REAGENT or RESOURCE	SOURCE	IDENTIFIER
**Chemicals, Peptides, and Recombinant Proteins**		

D(+)-Glucose monohydrate	Carl Roth GmbH + Co. KG, Karlsruhe, Germany	CAS-No: 77938-63-7
NaCl,	Merck KGaA, Darmstadt, Germany	CAS-No: 7647-14-5
KCl	Merck KGaA, Darmstadt, Germany	CAS-No: 7447-40-7
MgCl_2_	Merck KGaA, Darmstadt, Germany	CAS-No: 7791-18-6
CaCl_2_	Merck KGaA, Darmstadt, Germany	CAS-No: 10035-04-8
NaHCO_3_	Merck KGaA, Darmstadt, Germany	CAS-No: 144-55-8

**Deposited Data**		

Recorded spike times of direction-selective retinal ganglion cells under visual stimulation in-vitro	This paper	https://dx.doi.org/10.12751/g-node.0300fd

**Experimental Models: Organisms/Strains**		

Ambystoma mexicanum, adult pigmented wild-type, both sexes	Axolotl Facility, Center for Regenerative Therapies Dresden, Germany, and Adem Morankic, Reutlingen, Gemany	N/A

**Software and Algorithms**		

IGOR pro	WaveMetrics	RRID: SCR_000325
MATLAB	Mathworks	RRID: SCR_001622
Custom spike sorting algorithm in IGOR pro	adapted from Pouzat et al., (2002)	N/A
Custom analysis software in MATLAB	This paper	https://github.com/gollischlab/AnalysisForDSPopulationCodes

**Other**		

Multielectrode array workstation	Multichannel Systems, Reutlingen, Germany	USB-MEA256-System
Multielectrode arrays (252 electrodes; 30 μm electrode diameter; 100 μm minimal electrode distance)	Multichannel Systems, Reutlingen, Germany	256MEA100/30iR-ITO-w/o
OLED microdisplay	eMagin, Bellevue, Washington, USA	SVGA+ OLED-XL
Telecentric lens	Edmund Optics, Karlsruhe, Germany	2.0X SilverTL Telecentric Lens #58-431
Dialysis tubing cellulose membrane	Sigma-Aldrich, St. Louis, Missouri, USA	D9777-100FT
Image of leaves	McGill Calibrated Colour Image Database (http://tabby.vision.mcgill.ca/)	samplepippin0117.jpg
Image of pebbles	McGill Calibrated Colour Image Database (http://tabby.vision.mcgill.ca/)	samplepippin_Peel071.jpg

### Contact for Reagent and Resource Sharing

Further information and requests for resources and reagents should be directed to and will be fulfilled by the Lead Contact, Tim Gollisch (tim.gollisch@med.uni-goettingen.de).

### Experimental Model and Subject Details

Experiments were performed on 10 isolated whole-mount retinas from 7 healthy adult salamanders (Ambystoma mexicanum, pigmented wild-type, undetermined sex) of at least 12 months of age (exact ages unknown, birthdates not specified by supplier). Animals were housed on a 12-hour light-dark cycle in standard aquarium basins (1-2 animals per basin), equipped with burrow-like hide-out places and with constant water filtering, and were fed daily. Experimental procedures were in accordance with national and institutional guidelines and approved by the institutional animal care committee of the University Medical Center Göttingen (protocol number T11/35).

### Method Details

#### Electrophysiology

After an hour of dark-adaption, animals were sacrificed and eyes enucleated while keeping track of the eye’s orientation relative to the body as described previously (Kühn and Gollisch, 2016). Eyes were hemisected along the edge of the cornea, the vitreous humor removed, and the retinas separated from the eyecups while keeping track of their orientation. The pigment epithelium was peeled off, and the retina was mounted onto a semipermeable membrane, stretched across a circular plastic holder, with the photoreceptors facing the membrane. Membrane and retina were positioned onto a multielectrode array (MEA; Multichannel Systems, Reutlingen, Germany; 252 electrodes; 30 μm electrode diameter; 100 μm minimal electrode distance) such that retinal ganglion cells faced the electrodes of the MEA and upward motion of projected visual stimuli would later correspond to motion in the dorsal direction on the retina. Dissection and mounting were performed under infrared light on a stereo-microscope equipped with night-vision goggles. During recordings and dissection, the retina was superfused with oxygenated (95% O_2_, 5% CO_2_) Ringer’s solution, containing 110 mM NaCl, 2.5 mM KCl, 1 mM CaCl_2_, 1.6 mM MgCl_2_, 22 mM NaHCO_3_ and 10 mM D-glucose, pH 7.4, at a constant temperature of around 21°C. Voltage signals of retinal ganglion cells were recorded with 10 kHz sampling rate and band-pass filtered between 300 Hz and 5 kHz. Spikes were sorted offline with custom-made software, based on a Gaussian mixture model (Pouzat et al., 2002). As all animals in this study were from the wild-type group, no measures of experimenter “blinding” or randomization of subjects were applied. Experiments were replicated on multiple retinas (as reported in figure legends) with numerous individual recorded cells each. No prior sample-size estimation was performed.

#### Visual stimulation

Retinas were visually stimulated with a gamma-corrected monochromatic white OLED microdisplay (eMagin, Bellevue, Washington, USA) with 800 × 600 square pixels and 60 Hz refresh rate. Stimuli were projected through a telecentric lens (Edmund Optics, Karlsruhe, Germany) onto the retina. All projected stimuli were of 6.33 mW/m^2^ mean irradiance (low photopic light level), and pixels measured 7.5 × 7.5 μm^2^ on the retina. Stimuli were generated through custom-made software, based on Visual C++ and OpenGL.

### Quantification and Statistical Analysis

#### Receptive field properties

We used a spatiotemporal white-noise stimulus consisting of a checkerboard layout with 80x60 individual squares of 75 μm edge length to estimate the receptive field of a cell. Each square was randomly set to black or white (100% contrast) with a probability of 50% each and an update rate of 30 Hz. We obtained the spatiotemporal filter for each cell by calculating the spike-triggered average (Chichilnisky, 2001) and then used singular-value decomposition to separate the spike-triggered average into a spatial and a temporal receptive field component (Wolfe and Palmer, 1998). Cells with a low average firing rate for this stimulus (< 0.3 Hz) were not considered here. Spatial receptive fields were fitted with a two-dimensional Gaussian and represented by ellipses corresponding to the 1.5-SD contour of the Gaussian fits. The distance between the receptive fields of two cells was determined as the distance between the center points of the Gaussian fits.

#### Classification of standard direction-selective ganglion cells

We used drifting square-wave gratings of 100% contrast, 600 μm spatial period, and a temporal frequency of 0.75 Hz to identify direction-selective cells (Kühn and Gollisch, 2016). Gratings were shown in a sequence of eight equidistant directions with 6.67 s per direction, separated by 1.67 s of a gray screen (homogeneous illumination at mean intensity). The sequence was repeated five times. A direction-selectivity index (DSI) was calculated from the spike counts $f_{\theta }$ in response to the eight directions θ, leaving out the onset response to the first second of each direction, such that:

$$DSI=\frac{\left|\sum_{\theta }f_{\theta }e^{i\theta }\right|}{\sum_{\theta }f_{\theta }}$$

Cells with DSI>0.3 and a mean firing rate above 1 Hz for this stimulus were considered as direction-selective cells. The preferred direction of each direction-selective cell is then given by the angle of $\sum_{\theta }f_{\theta }e^{i\theta }$.

Furthermore, we focused on direction-selective cells that responded well to global motion rather than on object-motion-sensitive direction-selective cells, which represent a second class of direction-selective cells in the salamander retina (Kühn and Gollisch, 2016). To distinguish these cell classes, we applied a stimulus consisting of circular patches of 750 μm diameter, arranged in a hexagonal pattern on a mean-luminance background. The patches contained square-wave gratings of 300 μm period (*cf.* Kühn and Gollisch, 2016), which were jittered by selecting motion steps of 15 μm randomly to either side at 30 Hz. Stimulus segments of 23.33 s were presented repeatedly, separated by 1.67 s of gray screen, with all gratings jittering either coherently with the same trajectory or with independent, differential trajectories for each patch. An object-motion-sensitivity index $OMSI=\left(f_{d}-f_{c}\right)/\left(f_{d}+f_{c}\right)$ was calculated from the spike counts in response to coherent and differential motion, $f_{c}$ and $f_{d}$, respectively. Direction-selective cells with OMSI<0.7 and a mean firing rate above 1 Hz for this stimulus were considered not to be object-motion-sensitive and therefore to belong to the class of standard direction-selective cells (simply referred to as direction-selective cells in this work) and used for further analysis.

To determine which pairs of direction-selective cells had the same or different preferred directions, we first grouped the cells of each experiment into three groups based on their preferred direction (temporal, nasal-dorsal, and nasal-ventral, see Figure 2E) such that cells belonging to the same group or different groups were assumed to have same or different preferred directions, respectively. This was achieved by making use of the known orientation of the retina on the MEA and by determining the angle of the preferred direction relative to the nasal direction. Here, positive angles were defined in counterclockwise direction for the left eye and clockwise direction for the right eye when looking onto the photoreceptor side of the retina. Cells were then grouped into nasal-dorsal, temporal, and nasal-ventral groups, based on whether their preferred direction was between 0° and 120°, 120° and 240°, or 240° and 360°, respectively.

#### Contrast tuning

We tested the cells’ tuning to contrast by applying two full-field stimuli. First, we used flashes of 500 ms of increased or decreased light level at 40% from mean luminance, interleaved by 1.5 s of mean luminance. To assess the degree of ON versus OFF responses, an ON-OFF index was calculated from the spike counts $f_{on}$ and $f_{off}$, measured in a time window of 50 to 550 ms after the onset of the ON- and OFF-flash, respectively, such that the “Flashes ON-OFF Index” is $\left(f_{on}-f_{off}\right)/\left(f_{on}+f_{off}\right)$. Here, cells with a mean firing rate below 1 Hz for this stimulus were excluded, leading to 159 of 198 included cells from 10 retinas. Second, a temporal Gaussian-distributed white noise stimulus with standard deviation at 30% of mean luminance and 30 Hz update rate was used to calculate a spike-triggered average (STA). A “Flicker ON-OFF Index” was determined by summing the values of the last 200 ms of the STA prior to spiking and normalizing by the sum of the absolute values of the STA in this range, yielding an ON-OFF index in the range from −1 (pure OFF-peak) to 1 (pure ON-peak). Cells were only included if the absolute peak was seven times larger than the standard deviation of the STA tail (800 to 500 ms before spike), leading to 143 of 198 included cells. We furthermore assessed the contrast sensitivity by computing the nonlinearity of a linear-nonlinear (LN) model that takes the STA as the linear filter (details as in the analysis of motion trajectory encoding, see “LN model” below).

#### Complex texture motion

To probe the motion encoding of direction-selective cells for complex texture motion, we used a correlated noise texture, shifted in a two-dimensional random walk. The standard texture was generated from a white-noise pattern of black and white 30 × 30 μm^2^ squares (100% contrast) that was smoothed with a two-dimensional Gaussian kernel of 60 μm SD. The contrast values of the texture were then scaled by a factor of 1.5, with brightness values clipped at the maximum and minimum brightness of the screen. Textures were shifted every 33 ms in x- and y-direction with independent Gaussian-distributed motion steps of σ= 22.5 μm SD, which were rounded to multiples of 7.5 μm (the resolution of the screen).

In some experiments, we also applied two natural textures (Figure 3A) from the McGill Calibrated Color Image Database (Olmos and Kingdom, 2004), converted to grayscale by averaging the color channels (Liu et al., 2017), and a pink-noise texture with a 1/f spatial frequency spectrum. Pixel values for all textures were adjusted to the same mean intensity and SD as the standard texture and were shifted according to the same trajectory. In initial trial experiments, we also tested different motion step sizes and sizes of image blur. We found that with step size and blur reduced by a factor of two, direction-selective cells had low firing rates and could not provide information about the motion trajectory, probably due to their inability to resolve these fine spatial scales. Increasing step size and blur by a factor of two produced similar results in terms of correlations and information rates as obtained with the standard texture.

Mathematically, the motion trajectory can be denoted as a matrix

$$S=\left(\begin{array}{cc} s_{x1} & s_{y1}\\ s_{x2} & s_{y2}\\ \vdots & \vdots \\ s_{xM} & s_{yM} \end{array}\right)$$

of motion steps in x- and y-direction, $s_{xj}$ and $s_{yj}$, respectively, during time interval j of length Δt= 33 ms. M denotes the number of presented stimulus frames, depending on the length of the recording. The stimulus was usually presented for 30 to 40 min. In experiments where the effect of noise correlations was investigated, a 15-min trajectory was repeated 5 times. In experiments where we aimed at removing correlations from shared local contrast signals, the 15-min trajectory was repeated 7 or 9 times, with odd trials using the standard stimulus and even trials applying a texture layout that was translated by 1.5 mm in positive or negative x- or y-direction relative to the standard stimulus (*cf.* Figure 6D).

#### Directional preference under complex texture motion

In order to assess whether direction-selective cells responded in a direction-selective fashion to complex texture motion, we determined the average motion trajectory that elicited a response by calculating the spike-triggered average (STA) of the motion steps within an interval of LΔt= 800 ms prior to spiking (Chichilnisky, 2001). This is usually done by multiplying the sequences of motion steps with the spike counts of the following time bin and normalizing the summed sequences by the overall number of spikes. Equivalently, to facilitate later analyses, we here arranged the motion steps into a matrix $S_{seg}$ of trajectory segments of length L and binned responses in accordance with the motion steps, providing a response vector f:

$$S_{seg}=\left(\begin{array}{cccccc} s_{x1} & \cdots & s_{xL} & s_{y1} & \cdots & s_{yL}\\ \vdots & \; & \vdots & \vdots & \; & \vdots \\ s_{xM-L} & \cdots & s_{xM-1} & s_{yM-L} & \cdots & s_{yM-1} \end{array}\right),f=\left(\begin{array}{c} f_{L+1}\\ \vdots \\ f_{M} \end{array}\right)$$

where the elements $f_{j}$ (j=1,…,M−L) were the spike counts during time interval j+L of length Δt= 33 ms. The STA was then determined as the spike-count-weighted average of the trajectory segments, normalized by the total number of spikes

$$a=S_{seg}^{T}\cdot f/\sum_{j}f_{j}={\left(a_{xL}\;\cdots \;a_{x1}\;a_{yL}\;\cdots \;a_{y1}\right)}^{T}$$

where the $a_{xj}$ and $a_{yj}$ were the average motion steps in x- and y-direction, respectively, in the jth time interval prior to spiking. The preferred direction for complex texture motion was determined by summing the STAs in x- and y-direction, $v_{x}=\sum_{j}a_{xj}$ and $v_{y}=\sum_{j}a_{yj}$, resulting in a direction vector v. The angle of this vector then provided an estimate of the preferred direction.

Furthermore, we tested whether the motion STAs indicated a directional preference. Without direction preference, motion STAs should average out to zero, except for noise originating from limited sampling. We therefore compared the magnitude of the STA, given by the norm |a|, to the distribution of STA magnitudes of shuffled data, obtained by 1000 random shuffles of the spike times of the analyzed cell over the recording duration. An STA was considered significant if its magnitude was larger than 95% of the magnitudes from corresponding shuffled data.

#### Linear multi-cell decoder

We used a linear multi-cell decoder as introduced by Warland et al. (1997) to reconstruct the applied stimulus from the responses of a neuronal ensemble. In our case, the stimulus is given by the random motion steps in x- and y-direction, and responses are the spike trains of individual direction-selective cells (n=1) or of populations of up to n=30 direction-selective cells. First, spike counts $f_{j}^{i}$ of neuron i during time interval j were binned in accordance with the stimulus update rate and organized in a matrix, where each row contains the response sequences of all neurons for a time window of LΔt=800ms and different rows correspond to different, overlapping time windows:

$$F_{seg}=\left(\begin{array}{ccccccccc} 1 & f_{1}^{1} & f_{2}^{1} & \cdots & f_{L}^{1} & \cdots & f_{1}^{n} & \cdots & f_{L}^{n}\\ 1 & f_{2}^{1} & f_{3}^{1} & \cdots & f_{1+L}^{1} & \cdots & f_{2}^{n} & \cdots & f_{1+L}^{n}\\ 1 & \vdots & \vdots & \; & \vdots & \; & \vdots & \; & \vdots \\ 1 & f_{M}^{1} & f_{M+1}^{1} & \cdots & f_{M+L}^{1} & \cdots & f_{M}^{n} & \cdots & f_{M+L}^{n} \end{array}\right)$$

We calculated independent linear filters in x- and y-direction, $b_{x}$ and $b_{y}$, respectively, by convolving stimulus and response of the first 70% of the trajectory, $F{'}_{seg}^{T}\;\mathrm{S'}$, which was corrected by the pairwise correlations between cells, $F{'}_{seg}^{T}\;F{'}_{seg}$, resulting in a population filter

$$B={\left(F{'}_{seg}^{T}\;F{'}_{seg}\right)}^{-1}\cdot \left(F_{seg}^{'\;T}\;\mathrm{S'}\right)={\left(\begin{array}{ccccccccc} b_{x0} & b_{x1}^{1} & b_{x2}^{1} & \cdots & b_{xL}^{1} & \cdots & b_{x1}^{n} & \cdots & b_{xL}^{n}\\ b_{y0} & b_{y1}^{1} & b_{y2}^{1} & \cdots & b_{yL}^{1} & \cdots & b_{y1}^{n} & \cdots & b_{yL}^{n} \end{array}\right)}^{\text{T}}.$$

For cross-validation, a reconstruction of the motion steps in x- and y-direction $U=\mathrm{F'}{'}_{seg}\cdot B$ was determined from the responses $\mathrm{F'}{'}_{seg}$ to the remaining 30% of the stimulus. For illustrations, population filters were partitioned into their respective cell segments and the offsets $b_{x0/y0}$ were omitted (Figures 2A, 3B, 4E, and 8A–8D).

#### Mutual information

We estimated how much information the reconstruction provided about the actual motion trajectory by evaluating a lower bound of the mutual information $I_{s,u}=H_{s}-H_{s|u}$ between stimulus S and reconstruction U, analogous to Warland et al. (1997). Since motion in x- and y-direction were independent of each other, we performed this computation separately for x- and y-direction and then summed the two values to obtain a lower bound for the total information.

The Shannon entropy $H_{s}=-\sum_{s}p\left(s\right)\log_{2}\;p\left(s\right)$ is here based on the probability distribution p(s) of (non-overlapping) stimulus segments s in x- or y-direction, respectively, of length LΔt= 800 ms. The conditional entropy $H_{s|u}=-\sum_{u}p\left(u\right)\sum_{s}p\left(s|u\right)\log_{2}\;p\left(s|u\right)$ is derived from the probability of reconstruction segments p(u) (in x- or y-direction) and from the conditional probability p(s|u) of stimulus segments s given reconstruction segments u. The mutual information indicates how much the uncertainty about the stimulus S is reduced by knowledge of the reconstruction U. A lower bound of the mutual information follows from the fact that motion steps are normally distributed and that the reconstruction error e=s−u can be approximated by a Gaussian distribution with zero mean (Borst and Theunissen, 1999). The computation was performed in frequency space by obtaining the Fourier transforms, $\hat{s}$ and $\hat{e}$, of stimulus segments and reconstruction error segments, s and e, respectively. Then, the one-sided power spectra $P_{j}^{s}={\langle {\left|{\hat{s}}_{j}\right|}^{2}+{\left|{\hat{s}}_{-j}\right|}^{2}\rangle }_{seg}$ and $P_{j}^{e}={\langle {\left|{\hat{e}}_{j}\right|}^{2}+{\left|{\hat{e}}_{-j}\right|}^{2}\rangle }_{seg}$ were derived by averaging over all segments, ${\langle \cdot \rangle }_{seg}$, at each frequency j/(LΔt), 0≤j≤L/2. This yielded a lower bound of the information density $i_{s,u}^{j}>\log_{2}\left(P_{j}^{s}/P_{j}^{e}\right)$, which gives the number of bits/s/Hz that are provided within each frequency band (Figure 2C). The total mutual information for each motion direction was then calculated by summing across all frequency bands such that $I_{s,u}>\sum_{j=0}^{L/2}\log_{2}\left(P_{j}^{s}/P_{j}^{e}\right)/\left(L\text{Δ}t\right)\;$(Borst and Theunissen, 1999, Warland et al., 1997).

For comparing the information gain for different groups of neurons, an information ratio $I_{pop}/\sum_{k}I_{k}$ was derived from the mutual information of the reconstruction from the population responses, $I_{pop}$, and the summed mutual information from the single-cell reconstructions, $I_{k}$, within each population. For the analysis of pairs of direction-selective cells, we excluded pairs with a summed single-cell information below 0.1 bits/s (approx. 15% of the pairs) since the information ratios of these pairs are more susceptible to small fluctuations in their information estimates and mostly add noise to the data.

Pair information ratios were displayed in boxplots where boxes indicate the interquartile range (IQR, 25^th^ to 75^th^ percentile) and horizontal lines depict the median of the determined information ratios. Vertical lines extend to data points within 1.5 x IQR, and dots indicate outliers beyond this range. Since information ratios were usually not normally distributed, we used a nonparametric Wilcoxon signed-rank test to determine whether information ratios were significantly different from unity. For comparing information ratios between different conditions, we used a paired Wilcoxon signed-rank test.

#### LN model

To investigate the mechanisms underlying synergy in linear motion decoding, we extended the STA analysis to estimate a full linear-nonlinear (LN) model (Chichilnisky, 2001). To do so, we computed the filtered motion trajectory $g=S_{seg}\cdot a$, where the STA a was normalized to unit Euclidean norm and the stimulus to unit variance. We then estimated the model’s nonlinearity, which describes the relation between g and the evoked spike counts f by sorting the $\left(g_{j},f_{j}\right)$ value pairs by increasing g and binning them into 15 bins containing the same number of pairs. To obtain the nonlinearity N(g)as in Figure 5B, we determined the mean values within each bin.

We determined the degree of non-monotonicity of each cell’s nonlinearity by introducing a U-shape index $U=\left(N\left(g_{min}\right)-N\left(0\right)\right)/N\left(g_{max}\right)$, where $N\left(g_{min}\right)$ and $N\left(g_{max}\right)$ correspond to the leftmost and rightmost values, respectively, of the binned nonlinearity, while N(0) corresponds to the value of the central bin. U is close to unity when the nonlinearity is almost symmetric and strongly U-shaped, close to zero for a flat left tail, and negative when the nonlinearity is monotonically rising.

#### Spike-triggered covariance analysis and model simulations

Most observed nonlinearities displayed an offset of around 2 Hz mean firing rate. This relatively high offset originates from motion orthogonal to the preferred direction of a cell, which may trigger spikes through luminance-related activation. To determine the contribution of orthogonal motion to the offset, we performed spike-triggered covariance analysis (Schwartz et al., 2006). We computed the spike-triggered covariance matrix

$$STC=\sum_{j}f_{j}\left(s_{j}^{T}-a\right){\left(s_{j}^{T}-a\right)}^{T}/\sum_{j}f_{j}$$

where $s_{j}$ are the row vectors of $S_{seg}$ and $f_{j}$ the elements of the response vector f above, and performed an eigenvalue analysis. We then focused on the eigenvectors corresponding to the two largest eigenvalues, which most clearly stood out from the spectrum of eigenvalues (Figure S2A). We found that one of these eigenvectors closely matched the STA, whereas the other corresponded to a motion trajectory in an orthogonal direction. For both eigenvectors, we then calculated conditional nonlinearities (Samengo and Gollisch, 2013) by selecting only those stimulus segments for which the stimulus projection onto the other eigenvector was small $\left(-0.5<g_{j}<0.5\right)$ and then computing nonlinearities in the same fashion as described above for the LN model.

To study the effects of different nonlinearity shapes on motion decoding, we simulated a pair of direction-selective cells with an LN model, using either a one-dimensional (Figures 5F–5H) or a two-dimensional motion trajectory (Figure S2). For each simulated cell, we applied the experimentally determined motion filters of the direction-selective cells in Figure 5A to the trajectory and determined the response rates in each time bin by using either a monotonic or a U-shaped nonlinearity. The applied nonlinearities were obtained by least-squares fits of either an exponential, $N\left(x\right)=Ae^{Bx}$, or a non-monotonic function with offset, $N\left(x\right)=C+Ax^{2}e^{Bx}$, to the conditional nonlinearities in Figure S2B.

For the model with one-dimensional motion, the conditional nonlinearity of the second STC eigenvector (which corresponds to the cell’s STA) from the first cell was used. Taking the conditional nonlinearity (Figure S2B, blue lines) for the fit is more appropriate than using the full nonlinearity (Figure 5B) because the latter contains a larger offset that is caused by motion orthogonal to the preferred direction, whereas we here consider only one-dimensional motion. The conditional nonlinearity takes this into account by focusing on stimulus segments for which the projection of the orthogonal component was particularly small.

For the model with two-dimensional motion, spike rates of each cell were determined by filtering the two-dimensional trajectory with the cell’s first two STC eigenvectors and passing the filtered signals through their corresponding fitted nonlinearities,$\;N_{1}\left(g_{1}\right)$ and $N_{2}\left(g_{2}\right)$. Spike rates were then determined by summing over both components, such that $N\left(g_{1},g_{2}\right)=N_{1}\left(g_{1}\right)+N_{2}\left(g_{2}\right)$ (Figure S2C).

Finally, the spike count per stimulus frame was determined by a Poisson process with the rate given by the calculated response rates. The responses of such modeled direction-selective cell pairs were analyzed in the same fashion as the experimental data, that is, they were used to reconstruct a trajectory by determining population response filters and the mutual information between stimulus and reconstruction was calculated as above.

#### Conditional texture STAs

We used the linearly filtered trajectory g to divide the responses to texture motion into two groups: Responses to preferred motion trajectories, with $g_{j}>0.5$, and responses to non-preferred motion trajectories, with $g_{j}<-0.5$. For both groups, these responses were then used to calculate conditional spatiotemporal STAs to texture motion by averaging the 800-ms stimulus sequences preceding each spike. As expected from the spatiotemporal correlations in the texture stimulus, the obtained conditional STAs showed strong correlations in space and time, with successive frames looking nearly identical. For further analyses and for the graphical representations in Figure 5H, we therefore selected a single frame of the conditional spatiotemporal STA as its spatial component. Concretely, we used the frame at 200 ms prior to spiking because this time corresponds to the typical peak time of the motion STAs. To reduce the effect of incomplete cancelation of non-relevant stimulus parts, we estimated the non-informative spatial pattern by averaging over all stimulus frames and subtracted it from the conditional texture STAs. We excluded cells that did not have clear spatial structure in the corrected texture STAs by discarding cells whose absolute peak in the spatial component of the conditional STA was smaller than four standard deviations of the pixel values in this frame, leading to 102 of 198 considered cells.

To assess whether direction-selective cells responded to bright or dark contrast during preferred or non-preferred motion, the contrast values of the darkest and brightest pixel in the considered STA frame were added up and divided by the standard deviation of all pixel values, resulting in a contrast bias estimate for preferred as well as for non-preferred motion trajectories. To determine whether the location of dark spots in the conditional STAs was systematically related to a cell’s preferred direction, we obtained the direction of the vector that connects the minimum of the spatial component of the conditional STA for preferred motion to the minimum for non-preferred motion. We then determined the angular difference between this direction and the preferred direction of the cell as obtained from drifting gratings.

#### Canonical correlation analysis

We used canonical correlation analysis (CCA, Macke et al., (2008)) to determine how population responses were coupled to certain motion features. This method captured the most reliably encoded motion modes of the trajectory together with their correlated population activity. For direction-selective cell pairs with either the same or different preferred directions, stimulus and response were binned into Δt=33ms intervals as above, and the time course of both was observed in parallel for segments of $\tilde{L}\text{Δ}t=2\;s$. For CCA, stimulus and response were organized into matrices of stimulus segments ${\tilde{S}}_{seg}$ and response segments ${\tilde{F}}_{seg}$ from the responses of the two cells $f_{i}^{1}$ and $f_{i}^{2}$, respectively (see also above)

$${\tilde{S}}_{seg}=\left(\begin{array}{cccccc} s_{x1} & \cdots & s_{x\tilde{L}} & s_{y1} & \cdots & s_{y\tilde{L}}\\ \vdots & \; & \vdots & \vdots & \; & \vdots \\ s_{xM-\tilde{L}+1} & \cdots & s_{xM} & s_{yM-\tilde{L}+1} & \cdots & s_{yM} \end{array}\right),$$

$${\tilde{F}}_{seg}=\left(\begin{array}{cccccc} f_{1}^{1} & \cdots & f_{\tilde{L}}^{1} & f_{1}^{2} & \cdots & f_{\tilde{L}}^{2}\\ \vdots & \; & \vdots & \vdots & \; & \vdots \\ f_{M-\tilde{L}+1}^{1} & \cdots & f_{M}^{1} & f_{M-\tilde{L}+1}^{2} & \cdots & f_{M}^{2} \end{array}\right)$$

Then, the $\left(2\cdot \tilde{L}\right)\times \left(2\cdot \tilde{L}\right)$ cross-covariance matrix between stimulus and response segments $C_{sf}={\langle {\left(\tilde{s}-{\langle \tilde{s}\rangle }_{seg}\right)}^{T}\cdot \left(\tilde{f}-{\langle \tilde{f}\rangle }_{seg}\right)\rangle }_{seg}={\langle {\tilde{s}}^{T}\cdot \tilde{f}\rangle }_{seg}-{\langle \tilde{s}\rangle }_{seg}^{T}\cdot {\langle \tilde{f}\rangle }_{seg}$ was determined, where ${\langle \cdot \rangle }_{seg}$ denotes the average across segments, which are the row vectors $\tilde{s}$ and $\tilde{f}$ of ${\tilde{S}}_{seg}$ and ${\tilde{F}}_{seg}$, respectively. The cross-covariance matrix was then whitened by the covariance matrix $C_{s}\approx \sigma^{2}1$ of the stimulus segments and the covariance matrix $C_{f}$ of the response segments, so that $C=C_{s}^{-1/2}C_{sf}\;C_{f}^{-1/2}$. For finding the average stimulus patterns that were maximally correlated with the pair responses, singular value decomposition (SVD) was applied to the whitened cross-covariance matrix, $C=\mathrm{UD}V^{\text{T}}$. The column vectors $u_{k}$ and $v_{k}$ of the unitary matrices U and V then provided the k-th stimulus and response components, ${\tilde{a}}_{k}=C_{s}^{-1/2}u_{k}$ and ${\tilde{b}}_{k}=C_{f}^{-1/2}v_{k}$, respectively, where the first five components of two sample pairs are shown in Figures 6A and 6B. The diagonal entries $\rho_{k}$ of the diagonal matrix D denote the correlation coefficients between ${\tilde{S}}_{seg}\cdot {\tilde{a}}_{k}$ and ${\tilde{F}}_{seg}\cdot {\tilde{b}}_{k}$, corresponding to stimulus and response filtered with their k-th component vectors, respectively. This provides an order to the pairs of stimulus patterns and response segments.

#### Response correlations

We estimated the linear response correlations between direction-selective cell pairs by calculating the Pearson correlation coefficient of the binned cell responses, $f^{i}$ and $f^{j}$, with

$$r_{ij}=\frac{{\langle f^{i\;T}\cdot f^{j}\rangle }_{t}-{\langle f^{i}\rangle }_{t}^{T}\cdot {\langle f^{j}\rangle }_{t}}{\sqrt{{\langle f^{i\;T}\cdot f^{i}\rangle }_{t}-{\langle f^{i}\rangle }_{t}^{T}\cdot {\langle f^{i}\rangle }_{t}}\sqrt{{\langle f^{j\;T}\cdot f^{j}\rangle }_{t}-{\langle f^{j}\rangle }_{t}^{T}\cdot {\langle f^{j}\rangle }_{t}}}$$

For analyzing the correlations between the CCA response components, the Pearson coefficient was calculated from the upper and lower half of the k-th response component, ${\tilde{b}}_{k}^{1..L}$ and ${\tilde{b}}_{k}^{L+1..2L}$, respectively.

#### Subtractive and additive codes

Subtractive and additive codes of direction-selective cell pairs were determined by either subtracting or summing the binned spike counts of the two cells, resulting in a single response sequence. These response sequences were then used to calculate trajectory reconstructions, information estimates, motion STAs, and nonlinearities analogous to the single-cell analysis explained above. In addition, we computed information ratios for the subtractive code of direction-selective cells with different preferred directions by only considering motion along the spatial dimension (x- or y-direction) for which cells were motion-opponent. The appropriate dimension was selected as the one with the larger difference between the peak values of the two corresponding motion STAs in a 300 -ms interval prior to spiking.

### Data and Software Availability

MATLAB implementations of the linear decoder, mutual information measure, LN model, and canonical correlation analysis are available at:

https://github.com/gollischlab/AnalysisForDSPopulationCodes.

The data of this study are available at:

https://web.gin.g-node.org/gollischlab/Kuehn_and_Gollisch_RGC_spiketrains_for_moving_texture.

## Supplemental Information

Document S1. Figures S1 and S2

Document S2. Article plus Supplemental Information
