# Supplementary material for: Activity Correlations between Direction-Selective Retinal Ganglion Cells Synergistically Enhance Motion Decoding from Complex Visual Scenes
Source: Neuron. 2019 Mar 6;101(5):963–976.e7. doi: 10.1016/j.neuron.2019.01.003 (PMC6424814; doi:10.1016/j.neuron.2019.01.003)
Supplement: Document S1. Figures S1 and S2 [file mmc1.pdf]

Neuron, Volume *101*

Supplemental Information

Activity Correlations between Direction-Selective Retinal Ganglion Cells Synergistically Enhance Motion Decoding from Complex Visual Scenes

Norma Krystyna Kühn and Tim Gollisch

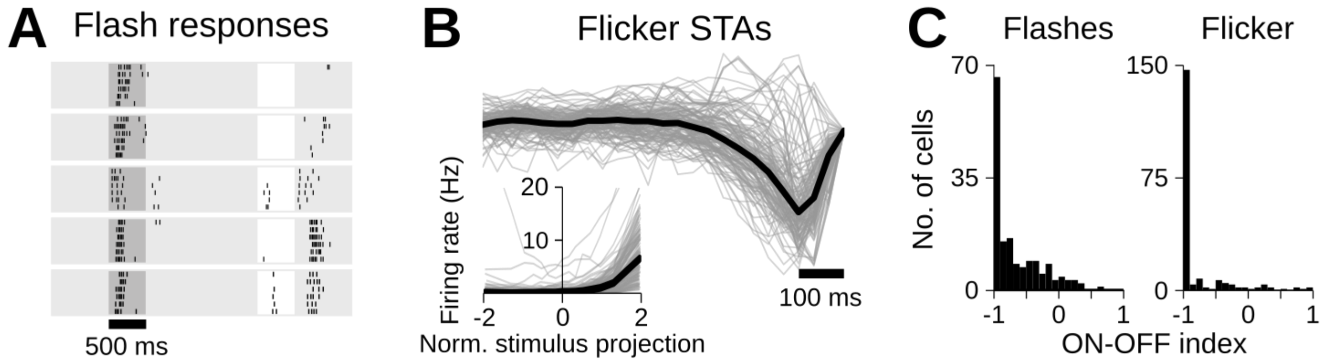

Figure S1: Direction-selective cells generally respond to OFF-type stimuli. Related to Figure 1.
(A) Direction-selective ganglion cells respond with robust spike bursts to step-like decreases in light level, but only weakly to light level increases. Raster plots for 5 direction-selective cells from 3 retinas to 6 repeated trials of OFF- (dark shaded areas) and ON-flashes (bright shaded) of 40% contrast from mean intensity (gray).
(B) Stimulation with white-noise flicker of light intensity reveals OFF-type response characteristics. Flicker spike-triggered averages (STAs) are dominated by negative peaks, and nonlinearities (inset) show a monotonic relation between stimulus contrast and response strength. Data of 143 direction-selective cells from 10 retinas. Black lines indicate mean over all cells.
(C) Histograms of ON-OFF indices from ON- and OFF-flashes ("Flashes", 159 cells from 10 retinas) and temporal white-noise flicker ("Flicker", 143 cells from 10 retinas) show strong preference for OFF-type stimuli. Positive range corresponds to ON-type responses, while negative range corresponds to OFF-type responses.

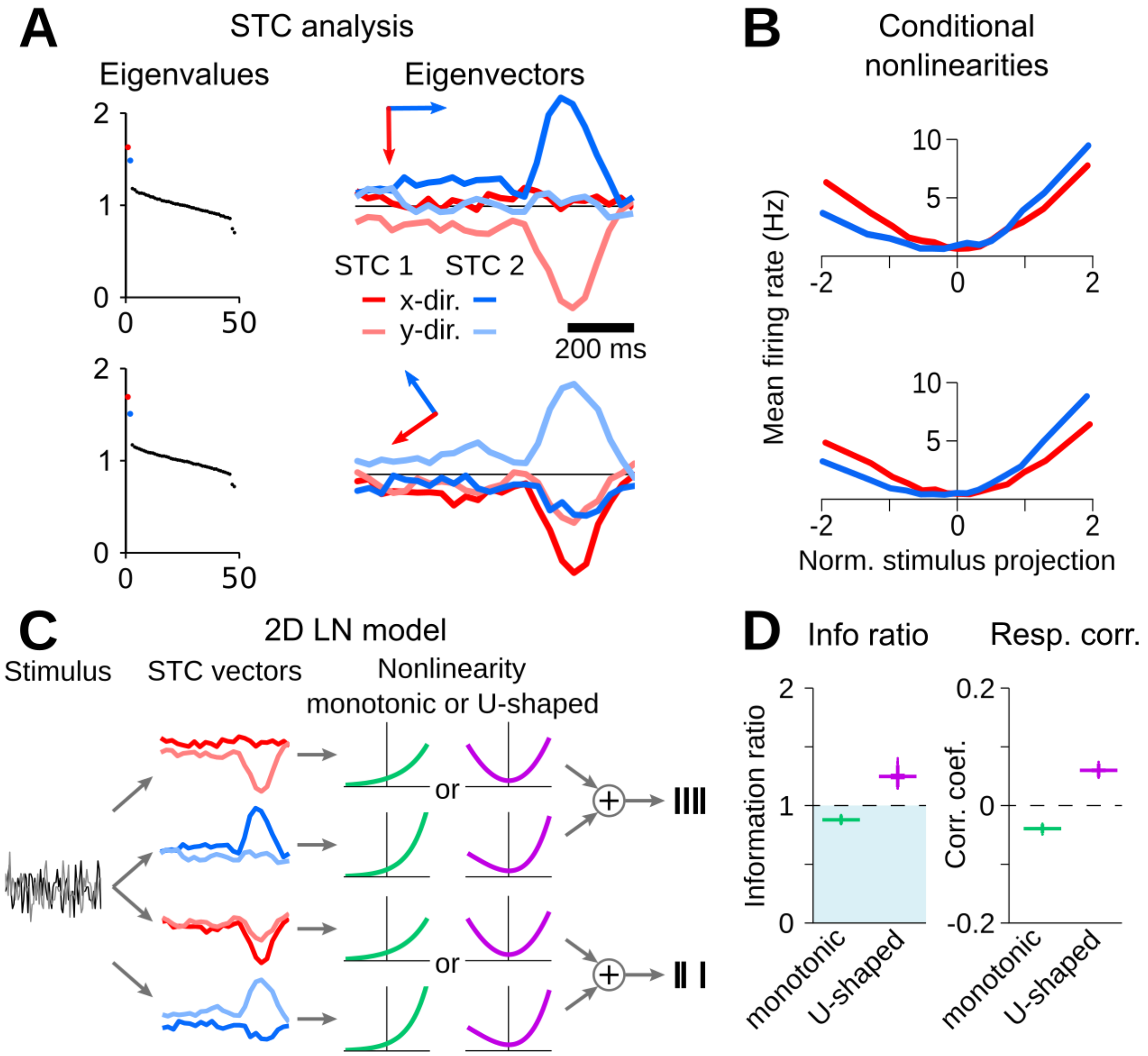

Figure S2: Two-dimensional LN model with U-shaped nonlinearities reproduces synergistic readout. Related to Figure 5 and STAR Methods.

(A) Spike-triggered covariance (STC) analysis provides a basis of orthonormal filters (eigenvectors) for the two-dimensional LN model. Eigenvectors of the two highest eigenvalues correspond to motion in the preferred direction of the cell (STC 2, blue) and motion orthogonal to it (STC 1, red). Example cells same as in Figure 5a. Blue and red arrows shown as insets indicate the motion directions in space corresponding to the eigenvectors, as obtained from taking the integrals of the eigenvectors (cf. Figure 1C).

(B) Conditional nonlinearities for the first two eigenvectors, obtained from stimulus segments for which the stimulus projection onto the other eigenvector was low (between -0.5 and 0.5).

(C) Schematics of two-dimensional model. For each cell, the two-dimensional trajectory is passed through two STC vectors, each two-dimensional, as obtained in (A). Then, either a monotonic (green) or a U-shaped nonlinearity (purple), which had been fitted to the conditional nonlinearities in (B), is applied. Resulting firing rates from the two eigenvectors were summed to yield a total firing rate. Spike counts were then determined by a Poisson process.

(D) Information ratios (left) and response correlations (right) from 1,000 repeated simulations of the two-dimensional LN model with different initializations of the Poisson process, each simulating 40 min recording time. U-shaped nonlinearities lead to a synergistic readout and positive response correlations, whereas monotonic nonlinearities do not.
